# Missed Perforated Appendicitis in a Nonverbal Patient with Autism and Duchenne Muscular Dystrophy: A Case Report

**DOI:** 10.3390/reports9040337

**Published:** 2026-10-07

**Authors:** Mona Makhlouf, Stanley Calderwood

**Affiliations:** 1 Keck School of Medicine, 1975 Zonal Ave, Los Angeles, CA 90033, USA; makhlouf@usc.edu; 2 Clinica Sierra Vista, 9525 Camino Media, Bakersfield, CA 93311, USA

**Keywords:** autism spectrum disorder, Duchenne muscular dystrophy, acute appendicitis

## Abstract

**Background and Clinical Significance:** A 4-year-old nonverbal boy with level 3 autism spectrum disorder and Duchenne muscular dystrophy (DMD) who was receiving chronic prednisone therapy presented to the emergency room with acute appendicitis. The diagnosis was significantly delayed due to atypical pain presentation, a potentially blunted inflammatory response from chronic steroid use, and an uncertain contribution from DMD-related muscle weakness. **Case Presentation:** The patient presented with five days of fever, poor oral intake, decreased stool output and behavioral changes. Initial physical examination and evaluation over two emergency department visits failed to establish the diagnosis. By the third visit, the patient exhibited abdominal distension, hypoactive bowel sounds, and bandemia. Computed tomography demonstrated perforated appendicitis with a walled-off abscess. Nonoperative management was unsuccessful, resulting in the development of multiple intra-abdominal abscesses that required percutaneous drainage. Following a 10-day hospitalization, the patient improved and was discharged. He subsequently underwent a scheduled laparoscopic appendectomy two months later, with pathology demonstrating persistent inflammation. **Conclusions:** This case raises awareness of the atypical presentation of pain in children with autism and the potential for DMD and chronic steroid therapy to complicate clinical assessment. These factors should prompt clinicians to maintain a low threshold for advanced imaging when clinical concern is present. It also illustrates the importance of close monitoring during nonoperative management of perforated appendicitis in medically complex or immunosuppressed children.

## 1. Introduction and Clinical Significance

Acute appendicitis is one of the most common abdominal emergencies and timely diagnosis is essential to prevent complications such as perforation and abscess formation. Diagnosis typically relies on history and physical exam findings, with the classic presentation including migration of pain from the periumbilical region to the right lower quadrant, vomiting, and fever [1]. In younger children, however, these classic features may be absent, contributing to delayed diagnosis [2,3].

Diagnostic challenges are amplified in children with autism spectrum disorder (ASD), particularly those who are nonverbal. Rather than verbalizing discomfort or localizing pain, these children may express distress through deviations from their baseline behavior, including increased irritability, aggression, self-injurious behaviors, or altered facial expressions [4]. Consequently, their behavioral manifestations are often misunderstood, leading to higher rates of complications from acute appendicitis [5,6]. Furthermore, commonly used visual self-report pain assessment tools like the Wong–Baker FACES Pain Rating Scale may have limited utility in pediatric populations with ASD or cognitive delays. This inaccuracy stems from developmental barriers that limit their ability to map their internal sensory experiences onto abstract pictorial representations of emotion [4,7].

Physical examination may be especially difficult in children with neurodevelopmental conditions who cannot reliably report symptoms, localize pain, or cooperate with abdominal palpation. Although Duchenne Muscular Dystrophy (DMD) causes progressive skeletal muscle weakness, the extent to which it alters peritoneal signs in young children is uncertain [8,9]. In addition, chronic corticosteroid therapy may increase susceptibility to infection and attenuate inflammatory responses, potentially complicating the recognition of appendicitis [10].

Herein, we present the case of a 4-year-old nonverbal boy with the coexistence of level 3 ASD and DMD on chronic steroid therapy whose atypical presentation resulted in a delayed diagnosis of acute appendicitis, leading to perforation and abscess formation. This case highlights the importance of maintaining a high index of suspicion for appendicitis in medically complex children who present with behavioral changes or nonspecific symptoms despite initially reassuring evaluations.

## 2. Case Presentation

A 4-year-old nonverbal boy with level 3 ASD and genetically confirmed DMD due to a deletion of exons 53–60, managed with prednisone 10 mg daily, presented to his pediatrician with a five-day history of fever, poor oral intake, decreased stool output, frequent crying and poor sleep. Serial vital signs, laboratory results, imaging findings, and key clinical events are summarized in Table 1.

Because he was distressed, combative, unable to follow examination instructions, and unable to communicate the presence or location of pain, focal tenderness and voluntary versus involuntary guarding could not be assessed reliably. His temperature was 38.3 °C, with otherwise normal vital signs. He was referred to the emergency department for further evaluation, where repeat temperature was 36.4 °C. Abdominal examination was reportedly benign, with normal bowel sounds and no tenderness, guarding or rigidity. His Wong–Baker FACES Pain Rating Scale was 0. Chest radiograph was normal. Despite the limited reliability of the examination, no focal abdominal findings were documented. He was diagnosed with presumed gastroenteritis and discharged with supportive care.

The following day, he returned to the emergency department due to persistent fever, irritability, poor oral intake, vomiting and four days without a bowel movement. His temperature was 38.9 °C and his Wong–Baker FACES Pain Rating Scale was 2. Repeat abdominal examination remained benign, demonstrating a soft, nondistended, nontender abdomen with normal bowel sounds and no organomegaly or palpable masses. Abdominal radiograph indicated a nonspecific bowel gas pattern with a mild stool burden and no free air or appendicolith. He was treated for constipation and discharged with instructions to continue supportive care.

**Figure 1:**
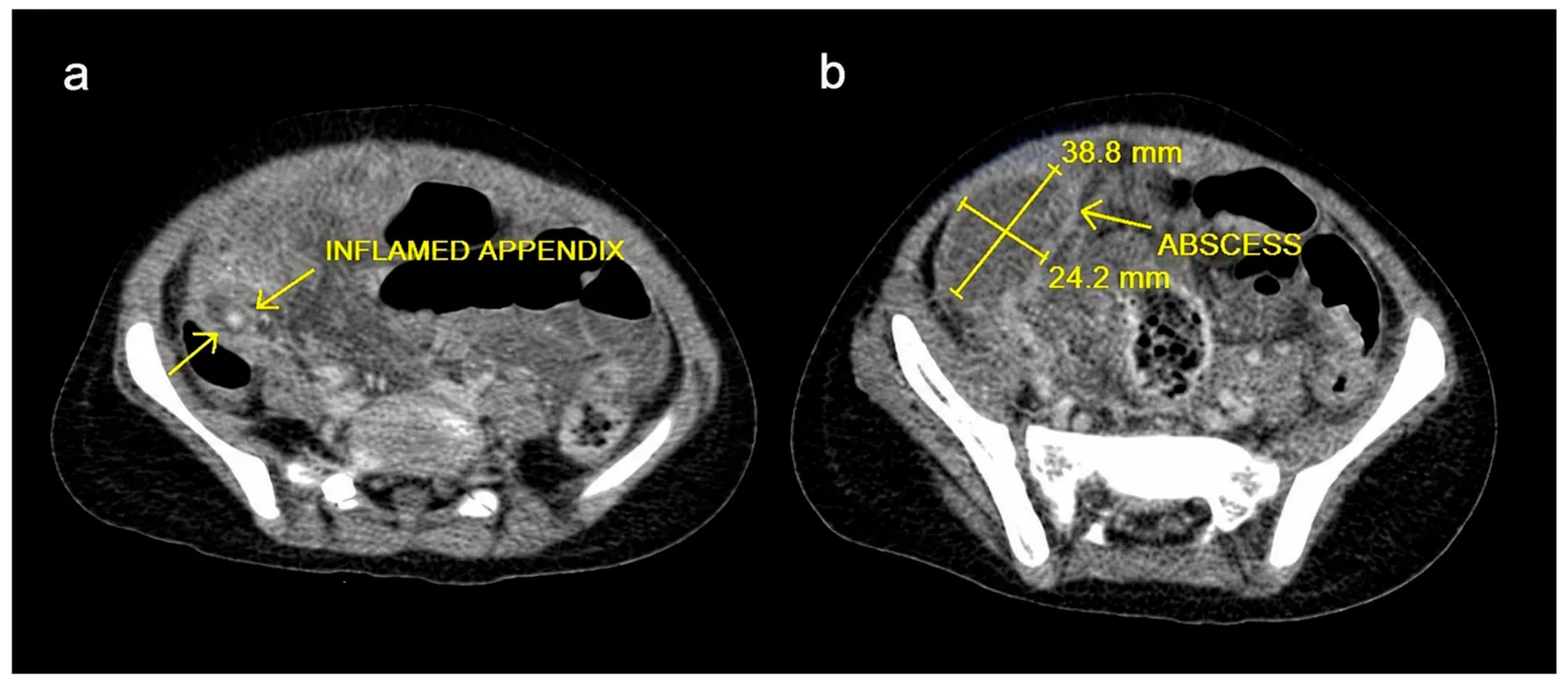
Axial contrast-enhanced computed tomography (CT) findings. (**a**) Inflamed appendix with surrounding periappendiceal inflammatory changes. (**b**) Periappendiceal abscess measuring 38.8 × 24.2 mm.

**Figure 2:**
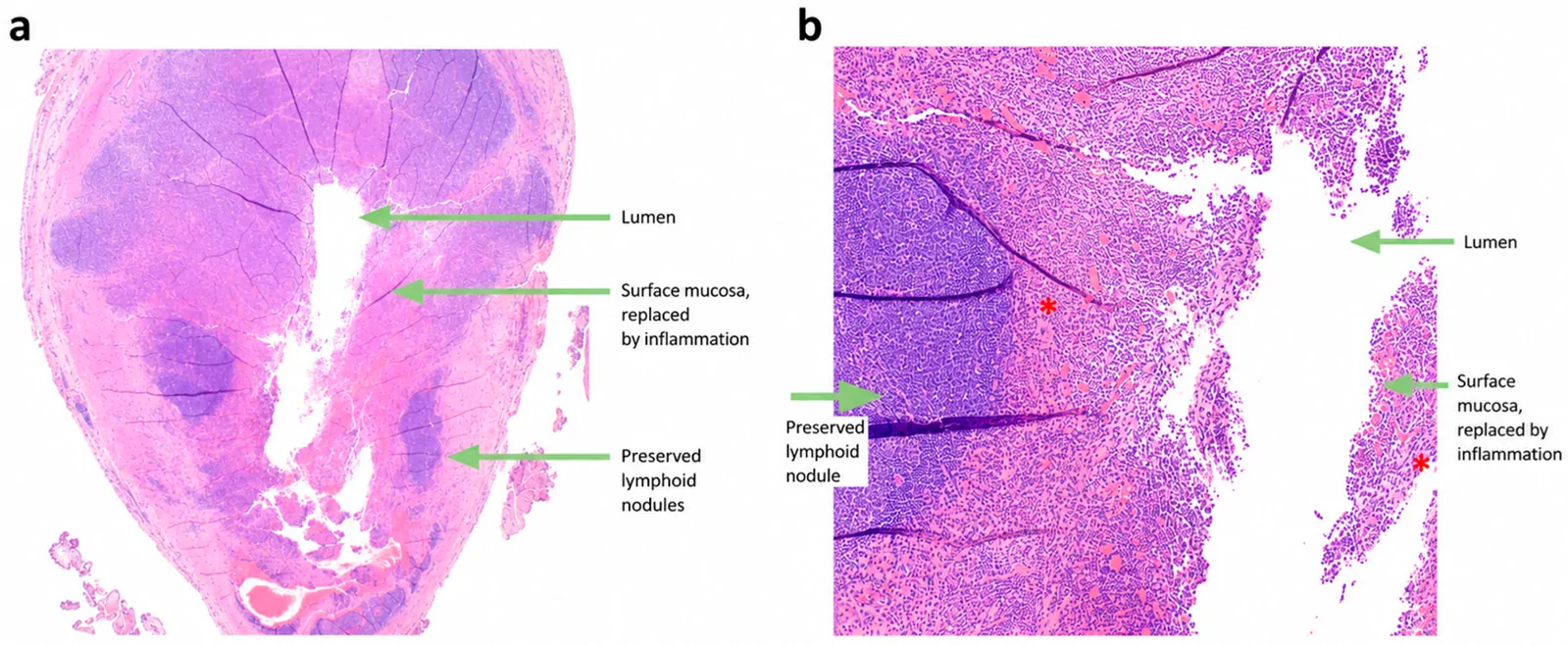
Cross-section of Appendix. (**a**) H&E stain at 2×. The entire surface mucosa has been replaced by inflammation; inflammation is seen spreading relatively close to the outer surface of the appendiceal wall; no residual appendiceal mucosa is present in this section. Nodules of residual normal lymphoid aggregates are present. (**b**) H&E stain at 20× demonstrating occasional multinucleate giant cells (*).

Two days later, he presented for a third time with persistent symptoms and worsening fever to 39.4 °C. Physical examination revealed abdominal distension and hypoactive bowel sounds. Computed tomography (CT) demonstrated perforated appendicitis with a walled-off abscess (Figure 1). He was admitted for nonoperative management with broad-spectrum antibiotics and supportive care. Four days after admission, progressive abdominal distension prompted abdominal ultrasonography, revealing multiple intra-abdominal abscesses. He subsequently underwent percutaneous drainage with catheter placement by interventional radiology and continued antibiotic therapy. The drains were removed four days later, and he was discharged after a 10-day hospitalization with an additional seven-day course of oral antibiotics. Two months later, he underwent an uncomplicated laparoscopic appendectomy, which showed persistent appendicitis with perforation (Figure 2).

## 3. Discussion

This case illustrates the potential diagnostic challenges arising when acute abdominal illness occurs in a young, nonverbal child with ASD, DMD, and chronic corticosteroid exposure.

Our patient lacked the classic history associated with acute appendicitis, including periumbilical pain migrating to the right lower quadrant and exacerbation of pain with movement. His inability to communicate or localize pain and the behavioral manifestations of distress complicated the clinical assessment. In this population, visceral pain frequently manifests as acute behavioral changes, such as heightened irritability, self-injurious behaviors, or aggression, along with poor cooperation with physical examination [4]. Caregiver interviews are therefore essential to establish the child’s behavioral baseline and identify meaningful deviations. Furthermore, clinicians should be aware that standard self-report pain assessment tools like the Wong–Baker FACES Pain Rating Scale can be difficult to interpret in neurodivergent children, who may have difficulty associating abstract pictorial expressions with their internal sensory experiences [7]. Despite the absence of classic localized pain findings, our patient had persistent fever, anorexia, vomiting, and decreased bowel output. Although nonspecific, the persistence of these findings across repeat visits warranted reconsideration for intra-abdominal pathology [3].

Our patient’s clinical course is consistent with prior studies reporting worse appendicitis outcomes in some children with neurodevelopmental conditions. Reeves et al. found increased odds of perforation among adolescents with ASD and increased odds of sepsis among children with ASD [6], while Lee et al. reported worse appendicitis outcomes among children with neurodevelopmental disabilities [5]. Our case illustrates potential contributors to these outcomes, including communication barriers, behavioral manifestations of pain, and limited cooperation with examination. However, as a single case, it cannot establish that ASD, DMD, or corticosteroid therapy independently caused the delayed diagnosis or perforation.

Classic peritoneal signs, including abdominal rigidity and guarding, were absent in our patient. The reliability of the abdominal examination was limited primarily by his inability to communicate or cooperate with the examination in the setting of level 3 ASD. Although coexisting DMD may theoretically have contributed through weakness of the abdominal wall musculature, causality cannot be established, the degree of abdominal wall weakness was not specifically assessed, and advanced muscular weakness would not typically be expected in a four-year-old. Therefore, the contribution of DMD to the examination findings remains uncertain. Nevertheless, secondary signs of peritonitis, including abdominal distension and hypoactive bowel sounds, were evident.

Chronic corticosteroid therapy may attenuate fever and leukocytosis while increasing susceptibility to infection, making appendicitis more difficult to recognize [10]. Although corticosteroid-induced demargination can complicate interpretation of the leukocyte count, bandemia remains an important indicator of acute bacterial infection, as was observed in our patient. Emerging evidence suggests that procalcitonin may be less affected by corticosteroid therapy than C-reactive protein and may therefore serve as a useful adjunctive biomarker for evaluating bacterial infection [10].

Plain abdominal radiography has limited diagnostic utility in suspected acute appendicitis and should not be used to exclude the diagnosis [11]. In our patient, the initial abdominal radiograph demonstrated only a nonspecific bowel gas pattern and mild stool burden, contributing little to the diagnostic evaluation. When clinical suspicion persists despite a reassuring physical examination or nondiagnostic radiographs, particularly in children with communication barriers or neuromuscular disorders, clinicians should maintain a low threshold for ultrasonography or CT.

Clinical prediction tools may support risk stratification for pediatric appendicitis, but their limitations are particularly relevant in nonverbal and neurodivergent children. The Pediatric Appendicitis Score (PAS) and pediatric Appendicitis Risk Calculator (pARC) incorporate subjective symptoms and examination findings—including pain migration, right lower-quadrant tenderness, and guarding—that may be difficult to assess reliably in children with communication differences [12,13]. Neither tool was developed specifically for this population, and the pARC was derived and validated in children 5 to 18 years of age, excluding children as young as the patient in this report [13]. Consequently, a low or equivocal calculated risk should not supersede caregiver-reported deviations from baseline behavior, persistent objective abnormalities, serial examinations, or worsening clinical status. An individualized pathway incorporating caregiver observations, repeated clinical and laboratory assessments, and early imaging may therefore be more appropriate than reliance on a clinical score alone. Once appendicitis is diagnosed, standardized severity classification may further support management decisions. The recently proposed WSES appendicitis grading framework links disease severity with relevant management considerations and may promote more consistent documentation, treatment decisions, and comparison of outcomes across future studies [14].

Nonoperative management with antibiotics is an accepted treatment strategy for perforated appendicitis with localized abscess formation [15]. However, careful patient selection and close clinical monitoring remain essential. In our patient, a localized abscess progressed despite antibiotic therapy, ultimately requiring percutaneous drainage of multiple intra-abdominal abscesses. This case illustrates that medically complex or immunosuppressed children undergoing nonoperative management may require escalation of care despite appropriate initial therapy.

Although this patient had ASD and DMD and was receiving prednisone, the diagnostic principles illustrated by this case may apply more broadly. Similar challenges may arise in children with intellectual disability, global developmental delay, cerebral palsy, severe communication disorders, or other conditions that limit their ability to report or localize pain. They may also arise in patients with neuromuscular weakness or immunosuppression that attenuates expected physical examination or inflammatory findings. Across these populations, clinicians should establish the patient’s usual behavioral and functional baseline, incorporate caregiver observations, and place greater weight on serial objective findings when conventional symptom reporting is limited.

## 4. Conclusions

This case highlights the potential diagnostic and management challenges of acute appendicitis in a nonverbal child with coexisting ASD and DMD who was receiving chronic corticosteroid therapy. Communication barriers, atypical pain behaviors, and attenuation of classic abdominal findings contributed to delayed recognition and subsequent perforation with abscess formation. Clinicians should maintain a high index of suspicion for serious intra-abdominal pathology in children with neurodevelopmental and neuromuscular disorders and consider early advanced imaging when symptoms persist or deviations from baseline behavior are present despite an initially reassuring evaluation. This case underscores the importance of close monitoring during nonoperative management of perforated appendicitis in medically complex children, as escalation of care may be required. Future research should include prospective, multicenter validation and recalibration of existing pediatric appendicitis risk tools in younger children and in children with communication, neurodevelopmental, or neuromuscular disorders. Diagnostic models could evaluate whether incorporating caregiver-reported deviations from baseline behavior, serial vital signs and examinations, laboratory trends, and ultrasound findings improves diagnostic accuracy. Multicenter registry studies and comparative-effectiveness studies could also examine imaging pathways and outcomes of operative versus nonoperative management in medically complex or immunosuppressed children, with stratification by standardized appendicitis severity grades.

## Figures and Tables

**Table 1:** Summary of Emergency Department Visits and Inpatient Management.

	11 June 2024	12 June 2024	14 June 2024	18 June 2024	23 August 2024
Temperature (Celsius)	36.4	38.9	39.4	36.8	-
Blood Pressure (mmHg)	-	99/68	-	109/85	-
Heart Rate (beats/minute)	134	159	-	150	-
Respiratory Rate (Breaths/minute)	24	22	24	22	-
Wong–Baker FACES Pain Rating Scale	0	2	-	-	-
White Blood Cell Count (WBC)	-	-	13.3 × 10^9^/L	-	-
Neutrophils (%)	-	-	48%	-	-
Band Count (as percentage of WBC)	-	-	17%	-	-
Anion Gap	-	-	17 mmol/L	-	-
Lactic Acid	-	-	1.0 mmol/L	-	-
Imaging	CXR ^1^	AXR ^2^	CT scan ^3^	Abdominal US ^4^	-
Diagnosis	Gastroenteritis	Constipation	Perforated appendicitis with abscess	Perforated appendicitis with multiple abscesses	Perforated appendicitis for interval appendectomy
Treatment	Ondansetron, ibuprofen and oral fluids	Ondansetron, ibuprofen alternating with acetaminophen	Interval appendectomy pathway	Percutaneous drain	Laparoscopic appendectomy
Pathology Report	-	-	-		Perforated vermiform appendix ^5^

^1^ No acute cardiopulmonary disease. ^2^ No free air, air-fluid levels or appendicolith identified. ^3^ Perforated appendix with walled-off abscess (see Figure 1). ^4^ Multiple intra-abdominal abscesses. ^5^ See Figure 2.

## Data Availability

The original contributions presented in this study are included in the article. Further inquiries can be directed to the corresponding author.

## Abbreviations

The following abbreviations are used in this manuscript:

ASD	Autism Spectrum Disorder
DMD	Duchenne Muscular Dystrophy
CT	Computed Tomography
pARC	Pediatric Appendicitis Risk Calculator
PAS	Pediatric Appendicitis Score
